# Induced diabetic neurogenic bladder animal models: application characteristics and standardization proposals via systematic data mining

**DOI:** 10.3389/fendo.2026.1804610

**Published:** 2026-07-07

**Authors:** Aihong Jin, Xiankang Shao, Xiaoping Liang, Bowen Xing, Simin Qin, Yujun He

**Affiliations:** 1 Department of Traditional Chinese Medicine, Taizhou Hospital of Zhejiang Province Affiliated to Wenzhou Medical University, Taizhou, Zhejiang, China; 2 Rehabilitation Department, The First People’s Hospital of Linhai City, Taizhou, Zhejiang, China; 3 Department of Preventive Treatment of Traditional Chinese Medicine, The First People’s Hospital of Chenzhou City, Chenzhou, Hunan, China; 4 Rehabilitation Medicine Department, The Second Affiliated Hospital of Hainan Medical University, Haikou, China

**Keywords:** data mining, detection markers, diabetic bladder dysfunction, diabetic neurogenic bladder, modeling parameters, preclinical animal model, validation criteria

## Abstract

**Background:**

Diabetic Neurogenic Bladder (DNB) is a prevalent urological disorder and autonomic nervous system complication associated with diabetes mellitus. This condition may lead to severe renal damage if not properly managed. Despite its clinical significance, there is a lack of unified standards for current DNB animal models, and previous review articles fail to provide sufficient objective data to support their conclusions.

**Objective:**

The present study aims to systematically summarize the characteristics of DNB animal models through data mining techniques, thereby offering a theoretical reference for the establishment of standardized DNB animal models.

**Method:**

Relevant studies were searched in 7 databases including PubMed and CNKI. After screening in accordance with the PRISMA flow diagram, a total of 352 eligible studies were included in the analysis. The SYRCLE tool was employed for quality assessment of the included studies, while SPSS Modeler (utilizing the Apriori algorithm) and R software were used for statistical analysis and result visualization.

**Results:**

SD rats, Wistar rats, and C57BL/6 mice were identified as the primary animal species used for constructing DNB models, with male animals accounting for 82.39% of the total. Chemical induction (predominantly streptozotocin, STZ; 74.15%) and combined induction with STZ plus a high-sugar and high-fat diet (13.64%) were the mainstream modeling methods. The most commonly used STZ dose was 60 mg/kg (administered as a single injection), and the typical modeling cycle was 8 weeks. The core criteria for successful modeling were the combined detection of blood glucose, urodynamics, and glycated hemoglobin (HbA1c). However, existing studies exhibited certain biases, such as insufficient blinding procedures. The characteristics of detection indicators were centered on a dual core of “basic pathological changes of diabetes mellitus + local bladder lesions,” involving mechanisms including inflammation, oxidative stress, fibrosis, neuroinjury, and cell death.

**Conclusions:**

This study clarifies the current application status of DNB animal models, addressing the research gap existing in previous review articles. Future research should focus on optimizing modeling protocols, promoting the application of non-invasive detection indicators, and enhancing the standardized reporting of study details.

## 1 Introduction

Diabetic neurogenic bladder (DNB), alternatively termed diabetic cystopathy, neuropathic vesicourethral dysfunction in diabetes, or diabetic bladder dysfunction, is recognized as one of the most prevalent autonomic neurological complications of diabetes within the disciplines of urology and endocrinology. With an incidence rate of up to 40%-60% among patients with diabetes mellitus, this condition is primarily featured by voiding dysfunction, which may further progress to upper urinary tract dilation, renal impairment, and even life-threatening complications in severe instances (1, 2). Notably, even strict glycemic control does not fully prevent the onset or progression of DND, underscoring the urgent need for reliable animal models to elucidate underlying mechanisms and develop targeted therapies (3–5). Contemporary therapeutic approaches encompass pharmaceutical interventions, pelvic floor rehabilitation training, neuromodulation techniques, and surgical procedures; nevertheless, the formulation of individualized treatment strategies remains a matter of controversy (6).

The pathogenesis of DNB involves a complex interplay of multiple factors, distinct from the core mechanisms of insulin deficiency and insulin resistance observed in diabetes. It primarily arises from structural and functional impairments affecting the bladder’s nerves, vasculature, and smooth muscle. These pathological changes are driven by a confluence of metabolic disturbances, oxidative stress, peripheral neuropathy, vascular injury, and inflammatory responses (7–9). Studies indicate that persistent hyperglycemia induces excessive generation of reactive oxygen species via NADPH oxidase activation, contributing to dysfunction of the bladder smooth muscle and neuronal damage (10). Additionally, autonomic neuropathy, particularly impaired parasympathetic function, results in diminished bladder sensation and weakened detrusor contractility. Dysregulation of the vascular endothelial growth factor signaling pathway further exacerbates the condition by potentially suppressing angiogenesis (11, 12). Recent evidence also highlights the significance of an imbalance between oxidative stress and neurotrophic factors, such as nerve growth factor and brain-derived neurotrophic factor, as a pivotal driver in the progression of DNB (13, 14).

Despite considerable research efforts dedicated to establishing animal models of DNB over the past decade, multiple feasible induction protocols have emerged; however, consensus on a universally standardized DNB model remains unrealized. Although prior review articles have cataloged existing DNB modeling frameworks, these syntheses frequently lack robust quantitative validation and omit precise specifications of essential experimental parameters—such as pharmacological dosing regimens, induction timelines, and model validation benchmarks (15). Data mining is formally defined as a computational methodology for extracting meaningful patterns and actionable insights from large-scale, incomplete, unstructured, and heterogeneous datasets (16). This approach has been increasingly adopted across biomedical research to decode latent structures within complex clinical data, thereby advancing strategies in disease prevention, diagnostic precision, therapeutic optimization, and evidence-based clinical decision-making (17, 18). Association rule mining is a data-driven approach designed to identify frequent patterns and co-occurrence relationships among categorical variables. Several algorithms are available for this purpose. The Apriori algorithm operates on a level-wise search strategy: it generates candidate itemsets and prunes infrequent ones using a minimum support threshold (19). FP-Growth, an alternative algorithm, bypasses candidate generation by constructing a frequent pattern tree, achieving faster processing speed on large datasets. However, FP-Growth is more memory-intensive and less straightforward to interpret when explaining how association rules are derived. For the present dataset, the Apriori algorithm offers distinct advantages. First, the dataset size is moderate, and computational efficiency is not a limiting factor; under such conditions, Apriori and FP-Growth produce comparable results (20). Second, Apriori’s iterative candidate generation and pruning process provides transparent, step-by-step interpretability—an important consideration for a methodology-focused manuscript aiming to inform future standardization of DNB modeling. Third, the Apriori algorithm is integrated into commonly used statistical software (e.g., IBM SPSS Modeler), enhancing the reproducibility of our analysis for other researchers in the field. Therefore, Apriori was selected for its balance of transparency, interpretability, and sufficient computational performance for the present dataset.

Building upon our team’s earlier data mining analysis of animal models for diabetic gastroparesis (21), the present study employs a systematic data mining framework to comprehensively integrate published literature on DNB animal models. Critical variables—including animal species and sex, induction methodologies, drug dosages, experimental durations, and validation criteria—are rigorously evaluated. Furthermore, we critically assess prevailing methodological inconsistencies and research gaps in current DNB modeling practices. The findings aim to deliver an evidence-informed foundation to guide the future development and standardization of reproducible DNB animal models.

## 2 Materials and methods

### 2.1 Data sources

A systematic data mining protocol was implemented to comprehensively aggregate and critically evaluate published studies concerning animal models of DNB and related diabetic lower urinary tract complications. Seven electronic databases were systematically queried: PubMed, Web of Science, Cochrane Library, China National Knowledge Infrastructure (CNKI), Wanfang Data Knowledge Service Platform, VIP Chinese Journal Database (VIP), and China Biomedical Literature Database (CBM). The search algorithm incorporated the following predefined terms in titles and/or abstracts: “diabetic neurogenic bladder”, “diabetic cystopathy”, “neurogenic vesicourethral dysfunction in diabetes”, and “diabetic bladder dysfunction”. No language or publication type restrictions were applied during initial screening. The search timeframe spanned from each database’s inception date to October 21, 2025. All retrieved records underwent standardized deduplication and eligibility assessment in accordance with the study protocol.

### 2.2 Eligibility criteria

Studies were eligible for inclusion provided they fulfilled all of the following criteria: (a) experimental studies demonstrating successful induction of DNB *in vivo*; (b) explicit documentation of essential modeling parameters, including animal species, sex, induction methodology, pharmacological dosing, experimental duration, and validation metrics; (c) full-text accessibility without language constraints; and (d) confirmation of ethical approval for animal procedures by an Institutional Animal Care and Use Committee or an equivalent institutional ethics committee.

### 2.3 Study exclusion standards

Publications were excluded if any of the following conditions applied: (a) incomplete, ambiguous, or unverifiable description of modeling methodology; (b) insufficient characterization of the DNB model or primary focus on non-urological diabetic complications; (c) non-original research outputs (e.g., review articles, commentaries, theoretical discussions, dissertations, conference abstracts, or clinical case reports); (d) duplicate records (in cases of bilingual publication, the English version was retained); and (e) exclusively *in vitro* investigations utilizing isolated cell cultures or tissue explants.

### 2.4 Data compilation and database development

Study selection followed the Preferred Reporting Items for Systematic Reviews and Meta-Analyses (PRISMA) framework (22). Retrieved records were imported into Zotero (version 7.0.30) for reference management and automated deduplication. Two independent reviewers (X. Shao and A. Jin) performed sequential screening of titles/abstracts followed by full-text assessment against predefined criteria. Discrepancies were resolved through consensus or adjudication by a third reviewer. Standardized data extraction covered species, sex, induction protocol, drug dosage, experimental timeline, validation criteria, and outcome measures. Extracted variables were systematically curated into a structured relational database using Microsoft Excel 2021. Cohen’s kappa coefficient was calculated to evaluate inter-rater consistency.

### 2.5 Methodological quality assessment

Risk of bias was evaluated using the SYRCLE (Systematic Review Centre for Laboratory Animal Experimentation) tool, a validated instrument comprising ten items across six domains specifically designed for preclinical animal studies (23). Each item was rated as “low risk,” “unclear risk,” or “high risk” based on predefined criteria: “low risk” indicated adequate bias mitigation strategies; “unclear risk” reflected insufficient methodological reporting; and “high risk” denoted features likely to compromise internal validity. SYRCLE remains the only tool explicitly developed for assessing bias in animal experimentation.

### 2.6 Quantitative data analysis

All itemsets were exclusively constructed for association analysis of model validation criteria. Only validation-related categorical detection indices formed core itemsets. Original continuous data on STZ dosage and modeling duration retained raw numerical values from included literature and were discretized strictly according to naturally concentrated numerical clusters observed in collected data, instead of artificially preset cut-offs; blood glucose grouping followed threshold standards originally adopted within included studies.

Association rule mining was performed using IBM SPSS Modeler (version 18.0), with supplementary analyses in Microsoft Excel 2021. The Apriori algorithm identified frequent itemsets and derived association rules related to DNB model induction parameters. Support denoted the joint occurrence frequency of antecedent and consequent itemsets; confidence represented the conditional probability of the consequent given the antecedent. Minimum support and confidence thresholds were iteratively optimized to ensure rule robustness and clinical interpretability, with procedural details referenced from prior methodological publications by our group (21). All visualizations were generated using R software (version 4.3.3).

## 3 Results

### 3.1 Literature screening and study inclusion

**Figure 1:**
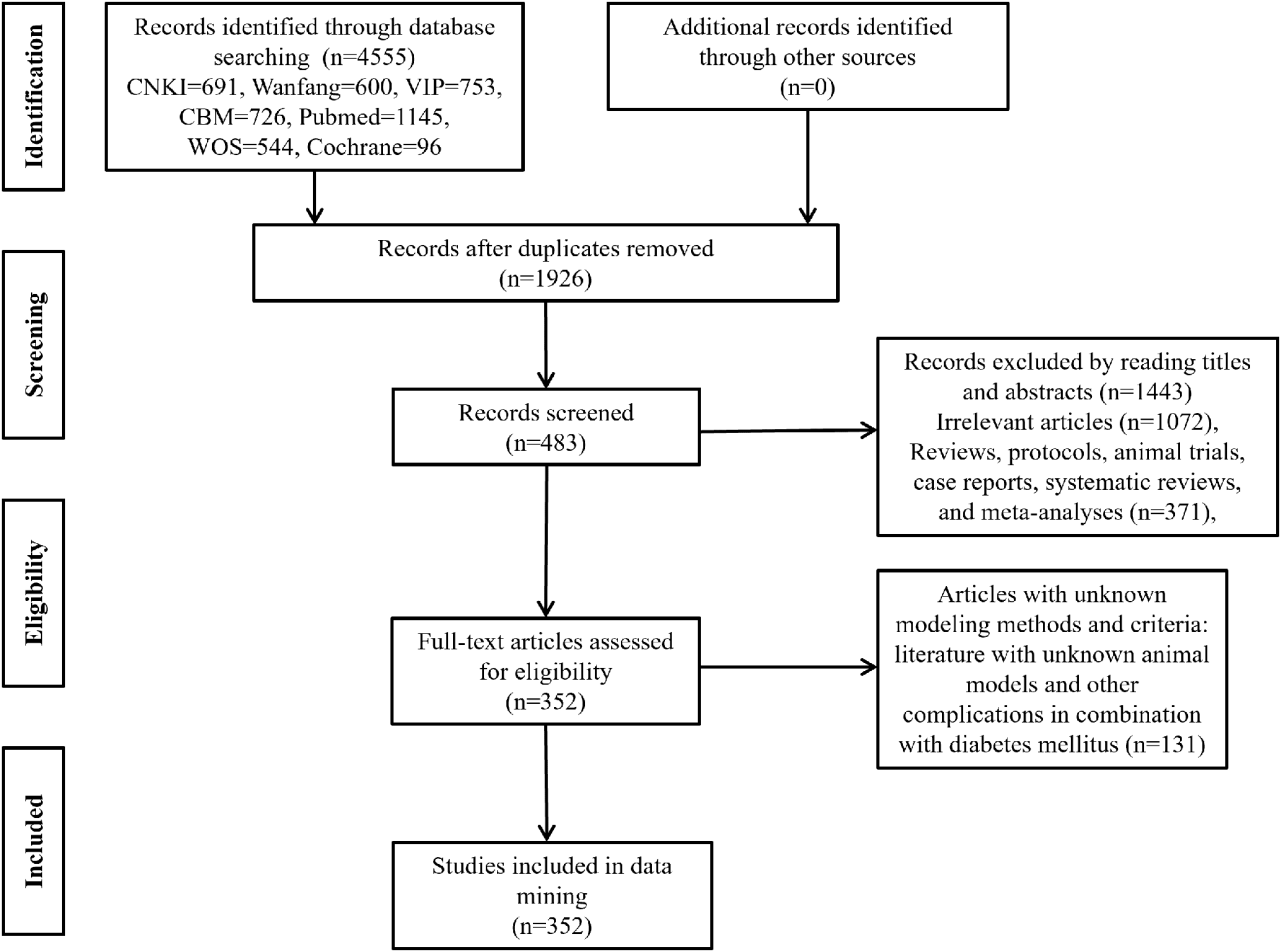
PRISMA flow diagram illustrating the process of literature identification, screening, eligibility assessment, and inclusion in the current systematic review.

The Cohen’s Kappa for full-text screening was 0.662, indicating moderate inter-rater consistency. All discrepant entries were settled by discussion or adjudication from a third investigator. A total of 4,555 studies were initially identified from the databases. After eliminating duplicate entries, 1,926 publications remained for further evaluation. An initial screening based on titles and abstracts led to the exclusion of 1,443 studies. Subsequently, a comprehensive full-text assessment was conducted applying pre-established exclusion criteria. Ultimately, 352 studies were included in the final analysis, while those lacking complete model-related data were omitted. The detailed selection process is summarized in **Figure 1**.

### 3.2 Assessment of methodological quality

The methodological quality of the 352 included studies was evaluated using the SYRCLE risk-of-bias tool, which indicated an overall acceptable risk of bias across studies. Among all evaluated items, only “blinding of experimentalists” showed a high risk of bias, accounting for 21.0% (74/352). The remaining items were predominantly associated with an uncertain risk, with no high-risk cases detected. In terms of bias categories, attrition bias (incomplete outcome data) and reporting bias (selective outcome reporting) exhibited the highest proportions of low risk, both at 62.2%. In contrast, “random housing”—a component of implementation bias—was associated with a low-risk rate of 48.9%. Over 74% of studies were rated as having an uncertain risk in the domains of “allocation concealation” (selection bias), “random outcome assessment,” “blinding of outcome assessors” (measurement bias), and “other bias.” Moreover, uncertain risk exceeded 50% for “sequence generation,” “baseline characteristics” (selection bias), and “blinding of experimentalists” (implementation bias). These observations underscore the need for subsequent studies to enhance blinding procedures for experimental operators and improve the reporting of methodological details to reduce uncertainties in quality assessment (**Supplementary Figure 1**).

### 3.3 Animal strains in DNB modeling

**Figure 2:**
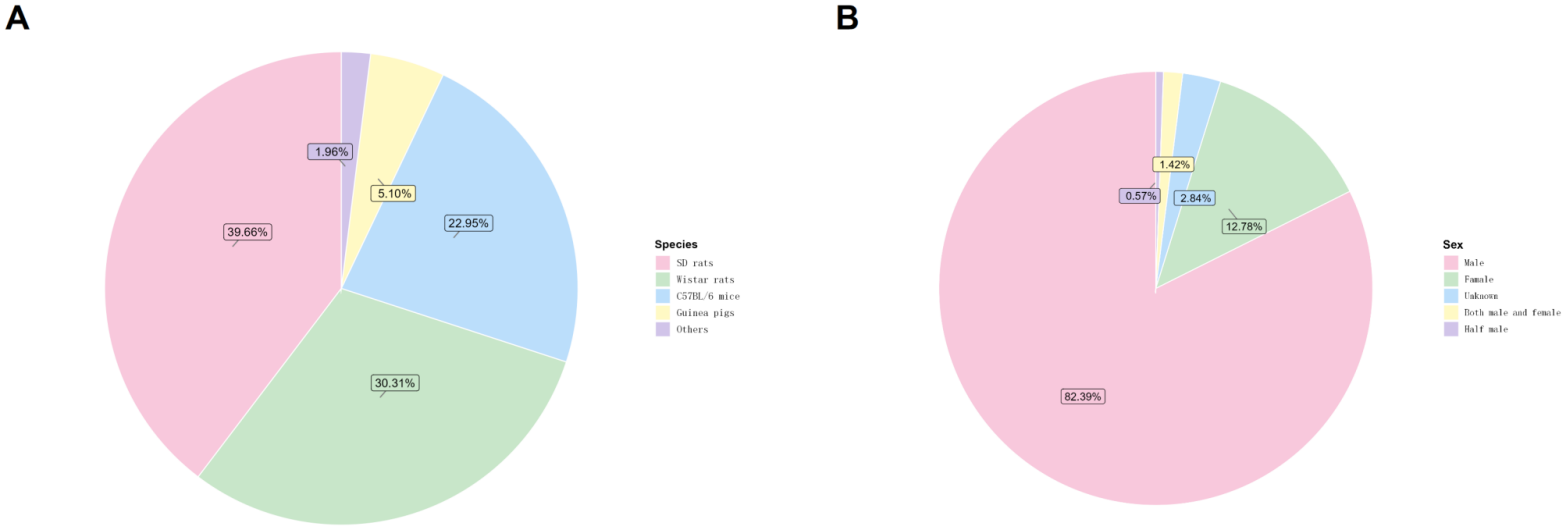
**(A)** Distribution of different animal strains employed in DNB modeling studies, with their corresponding frequencies. **(B)** Frequency distribution of animal sex (male, female, or other categories) used in DNB experiments.

Across the included studies, a total of 11 animal strains were employed for establishing DNB models, with some studies utilizing two strains concurrently. The analysis revealed that Sprague-Dawley (SD) rats were the most frequently used animals (140 occurrences, 39.66% of total applications), followed by Wistar rats (107 occurrences, 30.31%) and C57BL/6 mice (81 occurrences, 22.95%), as illustrated in **Figure 2A**. Only sporadic literatures reported genetically spontaneous diabetic strains including db/db mice and Zucker rats, accounting for less than 3% of total included experiments; therefore, quantitative statistical comparison between induced and inherited DNB models could not be performed in current analysis.

### 3.4 Sex distribution of experimental animals

Male animals constituted the predominant group used in DNB modeling (290 instances, 82.39% of total uses), while female animals were used in 45 instances (12.78%). The proportion of studies employing animals of other sex categories or combinations remained relatively low (**Figure 2B**).

### 3.5 Modeling methodologies

The modeling strategies adopted in the included studies were categorized into three types: chemical induction, combined induction, and dietary induction. Chemical induction was the most frequently applied method (264 instances, 75.00%), with streptozotocin (STZ) being the predominant agent (261 instances, 74.15%). Combined induction was employed in 85 instances (24.15%), most commonly involving STZ in conjunction with a high-sugar and high-fat diet (48 instances, 13.64%). Dietary induction alone was rarely used, appearing in only one study (**Supplementary Table 1**).

### 3.6 Dosage regimens in chemically induced models

**Figure 3:**
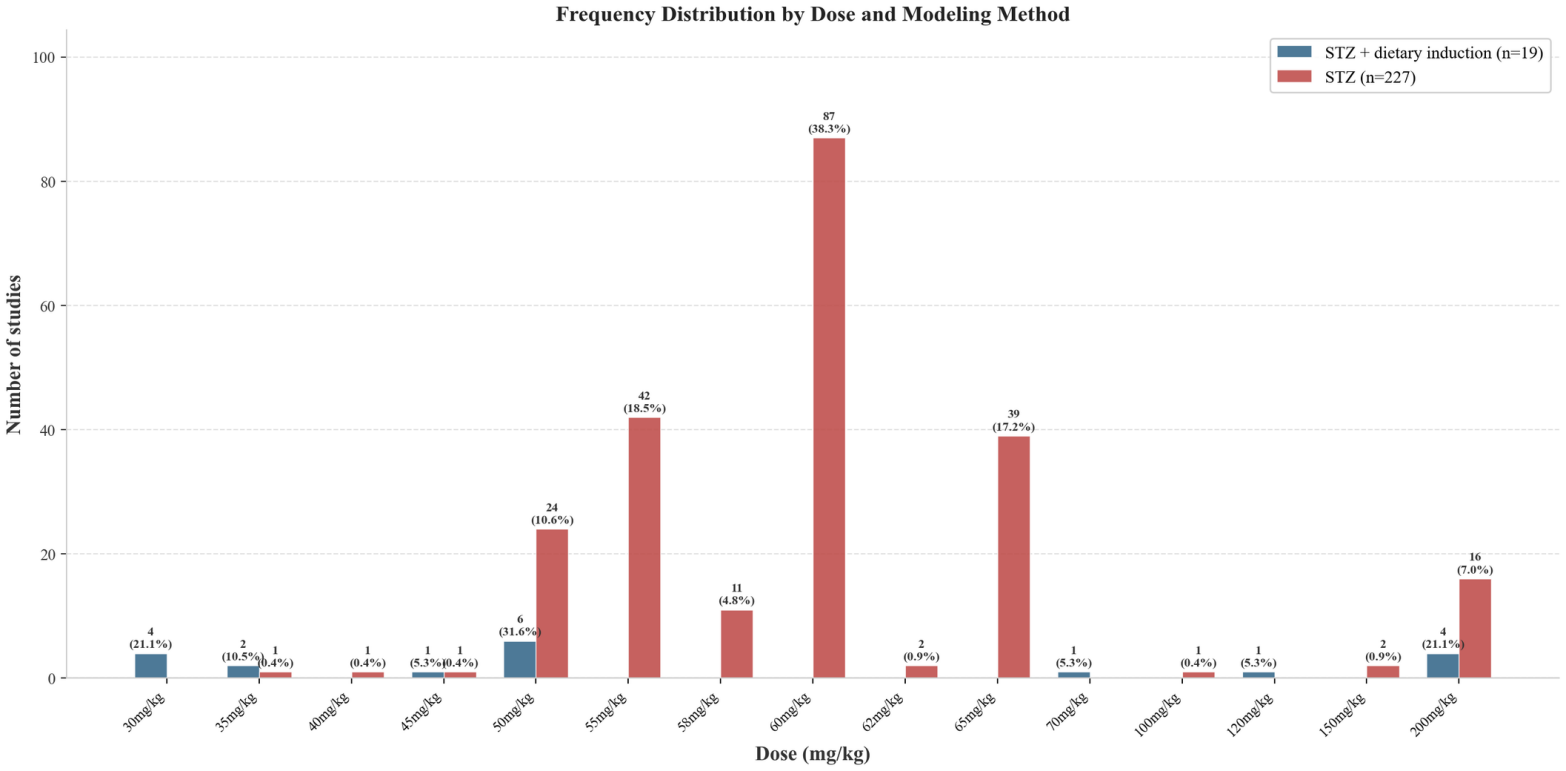
Dosage frequency of a single streptozotocin (STZ) injection across different DNB induction methodologies.

Given the widespread application of chemical agents for DNB induction, we systematically summarized dosing schemes across all chemical-induced protocols. Calculated from the total 347 chemically-induced experiments, single STZ 60 mg/kg ranked highest (87 instances, 25.07%), followed by 55 mg/kg (42 instances, 12.10%) and 65 mg/kg (39 instances, 11.24%). The dominant multi-injection STZ regimen was 50 mg/kg × five doses (24 instances, 6.92%). For STZ combined with high-sugar high-fat diet, single 50 mg/kg (4 instances, 1.15%) and repeated 30 mg/kg × three doses (10 instances, 2.88%) were most common. Alloxan was rarely adopted in only three studies (0.86%), uniformly given as a single 250 mg/kg bolus. Full dosage details are listed in **Supplementary Table 2**. Given the predominance of single-dose STZ injection among all chemical induction approaches, we further quantified the distribution of individual bolus doses, as illustrated in **Figure 3**.

### 3.7 Dietary induction strategies

To further investigate dietary modeling strategies, the specific nutritional protocols across all studies were compiled and analyzed. High-sugar and high-fat diet emerged as the most commonly used dietary intervention (49 instances, 56.98%), followed by high-fat diet (33 instances, 38.37%). High-sugar and high-salt diet accounted for a minor proportion. Ad libitum feeding was the predominant feeding method across all dietary models (**Supplementary Table 3**).

### 3.8 Modeling duration

**Figure 4:**
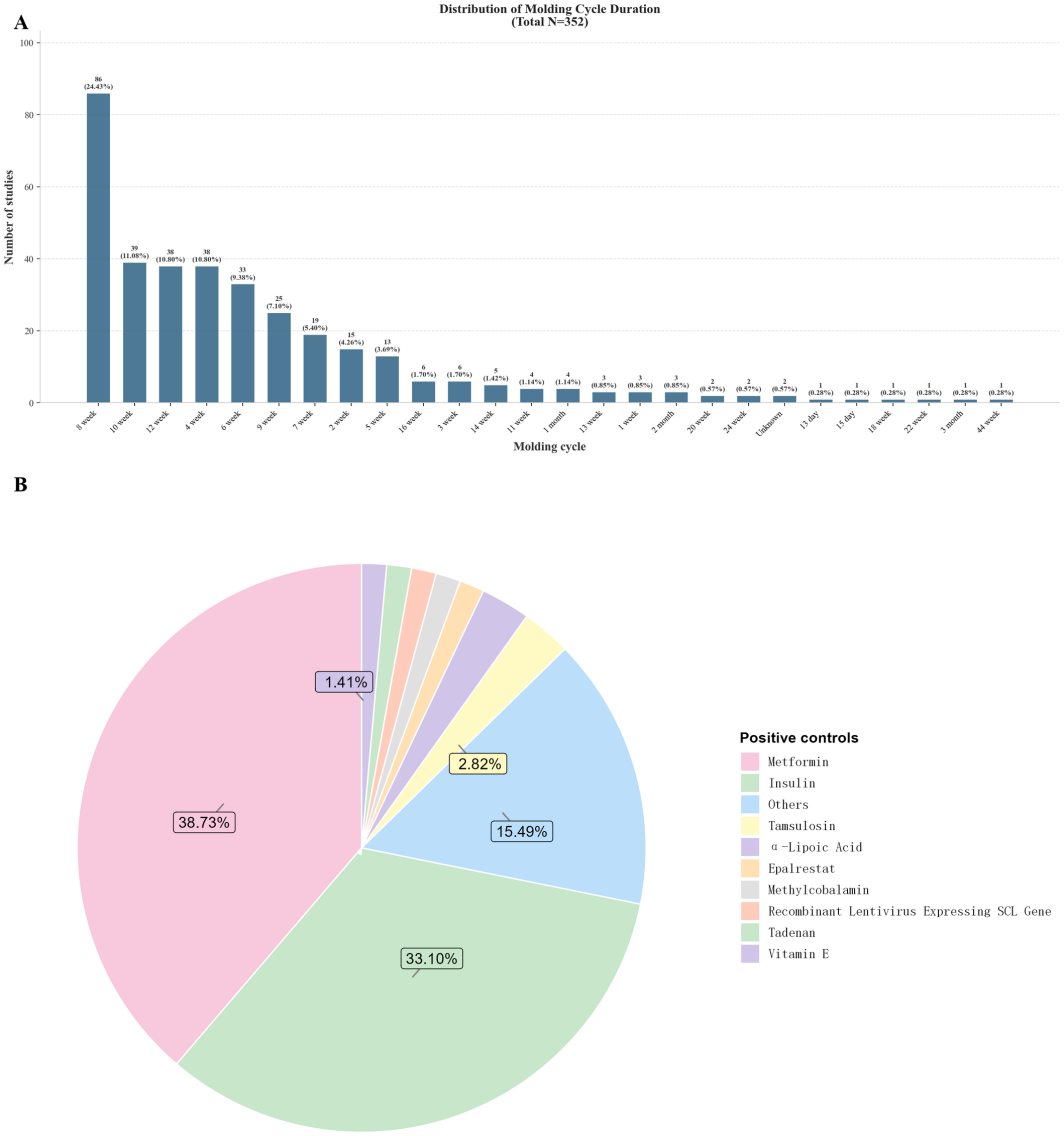
**(A)** Prevalence of various modeling durations (in weeks) adopted in DNB model establishment. **(B)** Frequency distribution of positive control agents applied in the included studies.

The time required for establishing DNB models varied considerably, largely due to the common practice of initial diabetes induction followed by dietary or natural progression. Two studies did not clearly report the modeling timeline. Among the remainder, 25 distinct modeling cycles were identified, ranging from 2 to 44 weeks. A cycle of 8 weeks was the most frequently reported (86 instances, 24.43%), as shown in **Figure 4A**.

### 3.9 Use of positive control agents

Among the 142 studies that employed positive control drugs, metformin was the most frequently used agent (55 instances, 38.73%), followed by insulin (47 instances, 33.10%), as summarized in **Figure 4B**.

### 3.10 Criteria for successful model establishment

**Figure 5:**
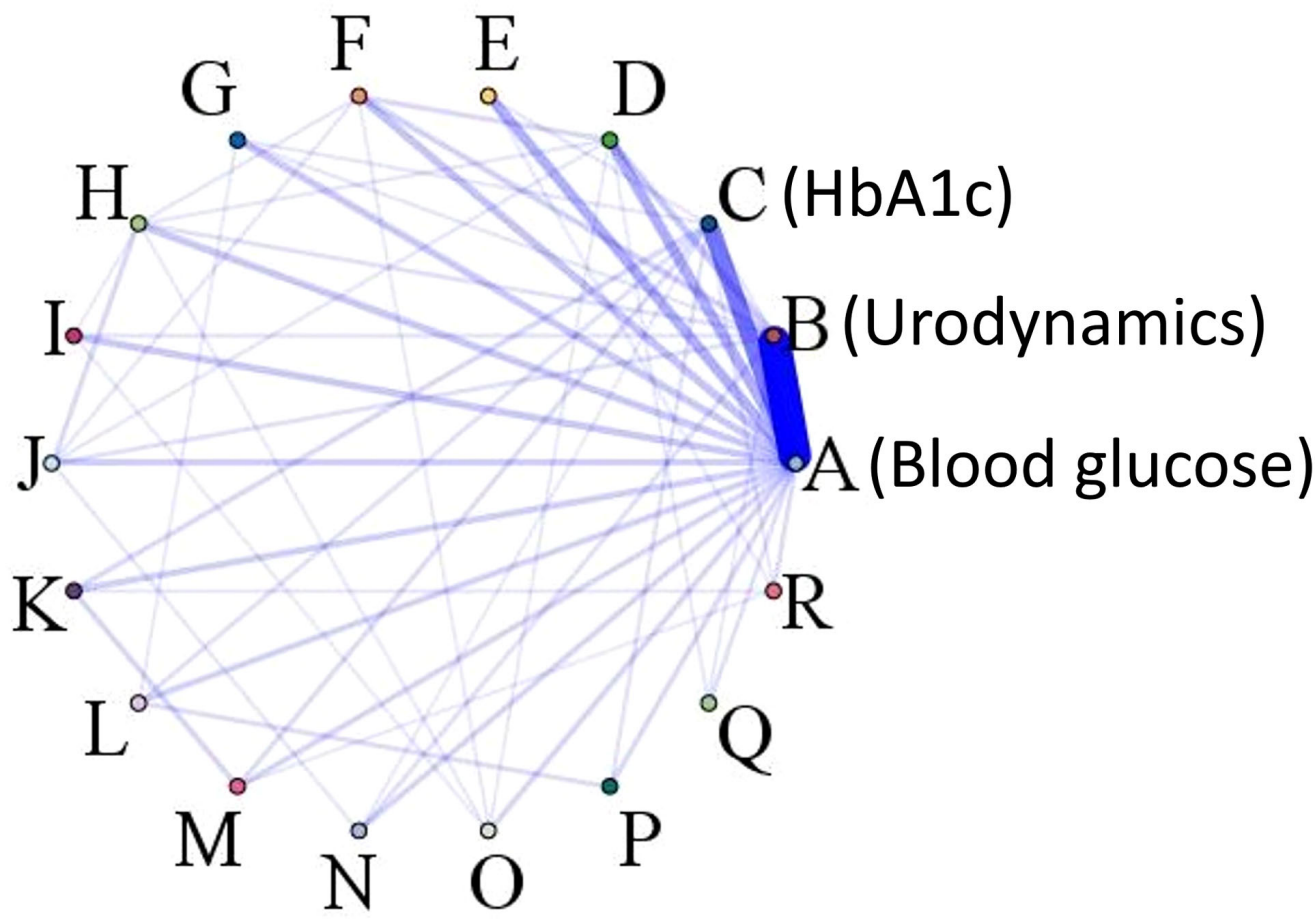
Network visualization generated from correlation analysis of key criteria used for evaluating successful DNB model establishment. **(A)** Blood glucose; **(B)** Urodynamics; **(C)** Glycated hemoglobin; **(D)** Bladder tissue pathology; **(E)** Insulin sensitivity; **(F)** Bladder wet weight; **(G)** Glucose tolerance; **(H)** General conditions; **(I)** Urine glucose; **(J)** Urine volume; **(K)** Urinary microalbumin; **(L)** Insulin; **(M)** Creatinine; **(N)** Voiding frequency; **(O)** Body weight; **(P)** Pancreatic β-cells; **(Q)** Isolated detrusor muscle experiment; **(R)** Renal tissue pathology. The thickness of the blue line represents the strength of the association between two elements, with thicker lines indicating stronger associations.

The criteria for determining successful DNB model establishment were categorized into several groups. Blood glucose parameters—such as fasting or random glucose levels exceeding thresholds like 11.1 (≈200 mg/dL) or 16.7 mmol/L (≈300 mg/dL)—were the most frequently applied (345 instances, 52.11%). Urodynamic assessments, including metrics such as maximum bladder pressure, bladder capacity, leak point pressure, and resting intravesical pressure, were used in 81 instances (12.24%). Glycated hemoglobin (HbA1c) criteria, defined by various cut-offs (e.g., ≥6.5%, >6.8%, ≥7.0%), were employed in 38 instances (5.74%) (**Supplementary Table 4**). Minimum support (3%) and confidence (50%) were finalized after repeated exploratory analysis. Support below 3% produced abundant trivial rare combinations lacking experimental reference, while a higher threshold discarded clinically valuable association rules; the 50% confidence cutoff eliminated weakly correlated associations. Notably, Apriori was only performed to mine correlations among model success evaluation indicators, not all extracted experimental parameters. The results indicated a strong association between blood glucose and urodynamic parameters (support = 23.01%, the 23.01% support of blood glucose + urodynamics combined detection equals 352×23.01%≈81 studies in total, meaning 81 out of all 352 articles simultaneously adopted these two indicators for model verification.), reflecting their frequent concurrent use (**Supplementary Table 5**). Network visualization further highlighted the synergistic relationship among blood glucose, urodynamics, and HbA1c—termed “A-B-C” association—suggesting that these three dimensions form a critical composite criterion for evaluating successful DNB model establishment (**Figure 5**). This outcome supports the validity of a multi-dimensional assessment system for DNB animal models.

### 3.11 Detection indicators

The outcome measures reported in the included studies were classified into 11 categories. The most frequently assessed indicators included blood glucose and insulin-related parameters (305 instances, 18.37%), histochemical staining (264 instances, 15.90%), and general condition observations (195 instances, 11.75%). A full breakdown is available in **Supplementary Table 6**.

## 4 Discussion

Diabetes mellitus has emerged as a critically important global public health challenge, marked by a consistent upward trend in prevalence worldwide over recent decades. This rise imposes significant pressures on healthcare systems and socioeconomic advancement across numerous countries (2, 24). Projections from epidemiological studies indicate that by 2030, around 643 million people—accounting for 11.3% of the global population—will be living with diabetes, and this figure is predicted to continue increasing (25). As a chronic metabolic disorder, diabetes contributes not only to elevated mortality and morbidity rates directly but also substantially heightens the overall disease burden due to a range of complications involving the urinary, nervous, and cardiovascular systems, as well as retinopathy (26). Among these complications, DND, which affects the urinary tract, is relatively common; however, its incidence is likely underestimated because it is frequently misidentified as other urinary system disorders (27). Importantly, tight long-term glycemic control in diabetic patients fails to completely block the initiation and progression of diabetic bladder dysfunction, even when blood glucose is maintained within optimal therapeutic ranges, which greatly highlights the urgent necessity to establish reliable preclinical animal models for pathogenic exploration and drug screening (3–5). Research utilizing animal models plays a crucial role in elucidating the pathophysiology of DND. Given ethical concerns and practical limitations in human-based research, animal models have become an essential means for exploring pathogenic mechanisms and potential treatment strategies for DND. Evidence indicates that diabetes-induced rodent models of neurogenic bladder can accurately replicate clinical pathological characteristics, including diminished bladder compliance, renal dysfunction, and bladder fibrosis, while also demonstrating a distinctive disease trajectory (28). Nonetheless, current approaches to DNB modeling are heterogeneous and lack consistent standards, highlighting the importance of further systematic synthesis using rigorous scientific methodologies.

The methodological quality assessment conducted with the SYRCLE tool highlighted several recurring limitations in current animal studies on DND. These concerns predominantly relate to incomplete reporting of key experimental details, which may compromise the validity, reproducibility, and interpretability of the findings. A. Inadequate Description of Randomization Procedures: The majority of studies omitted specific descriptions of the randomization techniques employed, often merely stating that “random grouping” was used. The absence of detailed methodology—such as the use of random number tables or computer-generated sequences—makes it difficult to verify whether proper randomization principles were followed. Furthermore, the lack of documentation regarding random sampling during outcome assessment introduces potential measurement bias. B. Absence of Blinding Measures: Blinding procedures were frequently not implemented for either the personnel administering interventions or those assessing outcomes. This omission can lead to performance and detection bias, potentially resulting in over- or underestimation of the intervention’s effects. When combined with inadequate randomization reporting, the absence of blinding significantly compounds biases related to estimating treatment efficacy. C. Limited Reporting of Baseline Animal Data: Critical baseline characteristics of the experimental animals—such as age, body weight, and health status—were often not reported. Given the frequently small sample sizes and inherent biological variability in animal studies, the lack of such information undermines the assessment of group comparability. This obscures the true intervention effects by failing to account for pre-existing differences, thereby weakening the reliability of the conclusions drawn. D. Insufficient Documentation of Housing Conditions: Key details regarding the animal housing environment—including temperature, humidity, light-dark cycles, and cage density—were not consistently provided. Inconsistent reporting of these environmental factors can lead to unintentional variations in experimental conditions, which may introduce performance bias and hinder the replication of studies. E. Incomplete Handling of Missing Data: Some studies reported instances of missing data or reductions in sample size but failed to explain the reasons for exclusions, describe methods used to handle missing values, or discuss the potential impact of missing data on the results. This practice raises concerns about selective outcome reporting and may threaten the internal validity of the study findings.

### 4.1 Animal selection and sex distribution in DNB modeling

In DNB research, animal models are selected based on genetic similarity to humans, physiological suitability, and practical experimental considerations. Rats, which share approximately 90% genetic homology with humans, are the most widely adopted species in DNB modeling. Among these, Sprague-Dawley (SD) rats are preferentially utilized due to their demonstrated lower mortality during model induction compared to Wistar rats, potentially attributable to reduced sensitivity to chemical modeling agents (29). While Wistar rats are also commonly employed, a minor subset may develop spontaneous diabetes in later stages, introducing an uncontrolled variable (30). Both SD and Wistar strains offer practical advantages, including low maintenance costs, rapid breeding cycles, and ease of handling. In contrast, C57BL/6 mice are used less frequently for urodynamic studies due to their smaller size, which complicates surgical procedures and metric assessments, and limits tissue availability for analysis. Although spontaneous models such as Zucker rats provide valuable insights into natural disease progression and mechanistic pathways, their application is constrained by extended development timelines and higher associated costs. Therefore, model selection should be guided by specific research objectives, weighing factors like feasibility, reproducibility, and pathophysiological relevance to ensure experimental validity.

Male rats constitute the predominant sex used in DNB modeling, a preference supported by several physiological and methodological factors. Anatomically, male rats typically possess a larger bladder capacity than females, facilitating more consistent urodynamic measurements (31, 32). The hormonal environment also plays a critical role; the testosterone-to-estradiol ratio is recognized as a key regulator of bladder function (33). The estrous cycle in females introduces hormonal fluctuations that can influence bladder sensitivity and contractility, thereby increasing experimental variability. In contrast, the more stable hormonal profile of male rats minimizes this confounding influence, enhancing the reliability of outcome data. Furthermore, the established historical precedent of using males in foundational DNB studies promotes consistency across the literature, enabling more robust cross-study comparisons and meta-analyses. Nonetheless, female models are essential for investigating sex-specific aspects of DND, such as the protective role of estrogen in bladder function (34) or the efficacy of therapeutic interventions in female subjects. Thus, the choice of animal sex should be deliberately aligned with the specific scientific question under investigation.

### 4.2 Modeling substances and methods

The STZ-induced method represents the most widely employed approach for establishing animal models of type 1 diabetes mellitus (T1DM) and is frequently utilized in subsequent studies aimed at inducing DND. STZ is a nitrosourea compound whose molecular structure resembles that of glucose, allowing it to selectively enter pancreatic β−cells via the glucose transporter 2 (GLUT2). Due to the high abundance of GLUT2 in β−cells and its limited expression in other cell types, STZ exhibits targeted cytotoxicity. Upon cellular uptake, STZ promotes β−cell damage through dual pathways: nitric oxide-mediated toxicity and oxidative stress. Given that pancreatic β−cells are the sole source of insulin, their impairment results in an absolute insulin deficiency, leading to disrupted glucose uptake and utilization, persistent hyperglycemia, and ultimately the manifestation of T1DM (35). It should be noted that STZ has been shown to directly activate the NLRP3 inflammasome in the bladder urothelium, which could provoke inflammatory bladder dysfunction independently of hyperglycemia. This direct urothelial toxicity represents a potential confounder that may partially uncouple bladder pathology from the diabetic state per se, and should be considered when interpreting mechanistic findings derived from STZ-induced models (36). An alternative chemical inducer, alloxan, has occasionally been used in DNB modeling. Alloxan can form a C5 gem−diol structure *in vivo*, generating high levels of reactive oxygen species (ROS) that selectively damage β−cells. However, alloxan is chemically less stable, shows considerable inter−animal variability, and is associated with nephrotoxic side effects, which collectively limit its application in contemporary research (37).

The regimen of STZ administration significantly influences the pathological changes in pancreatic islets. High−dose STZ injection (e.g., ≥60 mg/kg as a single dose) typically causes rapid and extensive loss of β−cell function and pronounced structural damage to islets (38). In contrast, repeated low−dose protocols not only mitigate acute toxicity but also sustain stable hyperglycemia while preserving a subset of β−cells, though this approach requires a longer induction period. Accordingly, different dosing strategies can simulate distinct types of diabetes: high−dose STZ is primarily used for T1DM−associated DND, whereas moderate doses (40–55 mg/kg) are often employed to mimic T2DM−related DND.

Combined induction using STZ and dietary manipulation is another widely adopted strategy. In this paradigm, dietary intervention (e.g., high−sugar/high−fat diet) induces insulin resistance, while low−dose STZ causes mild β−cell injury. Together, these factors synergistically elicit type 2 diabetes mellitus (T2DM), which closely mirrors the etiology of clinical DNB patients. Notably, T2DM accounts for more than 90% of diabetes cases (39), and the combined model recapitulates a disease progression similar to that in humans. Drawbacks include a prolonged modeling cycle, higher costs, and the need for strict control over dietary composition.

However, a critical question remains: do high-dose STZ models and low-dose STZ plus high-fat diet models produce fundamentally different urodynamic or histological DNB phenotypes? The present data mining analysis attempted to address this question but encountered several obstacles. First, a few the included studies explicitly classified their model as T1DM-associated or T2DM-associated DND. Second, among studies that did provide such classification, urodynamic protocols varied substantially (e.g., different anesthesia regimens, catheterization techniques, and outcome parameters), making direct cross-comparison difficult. Third, the available literature suggests that bladder hypertrophy is consistently observed in STZ-induced models but less consistently in T2DM models, even when blood glucose elevations are comparable. This observation hints that STZ may exert bladder-specific effects beyond hyperglycemia (e.g., direct NLRP3 inflammasome activation), potentially confounding the attribution of bladder pathology solely to T1DM pathophysiology. Therefore, the current evidence base does not permit a definitive conclusion regarding phenotype divergence between T1DM- and T2DM-associated DNB models. Future studies should systematically characterize urodynamic and histological endpoints in parallel T1DM and T2DM models using standardized protocols.

Spontaneous models, such as animals with leptin receptor deficiencies, develop insulin resistance, obesity, and T2DM naturally, and progressively exhibit DNB in later stages. These models require no exogenous drug administration, show high pathological consistency, and mimic the natural history of human disease, allowing long−term observation of disease progression. However, their high cost, demanding housing conditions, and extended breeding cycle have limited their widespread adoption. In summary, the choice of an appropriate modeling strategy should carefully balance the properties of the inducing agent, dose−response characteristics, and specific research objectives.

### 4.3 Temporal progression in DNB model development

The development of DNB models generally requires prior induction of diabetes, as lower urinary tract dysfunction manifests progressively with disease advancement. Consequently, experimental evaluations are typically conducted after a predetermined interval following the administration of the inducing agent to ensure full pathological development. The duration required for DNB establishment is variable; a window of 4 to 10 weeks is frequently adopted, with 8 weeks representing the most commonly selected time point.

Studies have shown that during the initial phase (0–4 weeks), the bladder undergoes a compensatory stage characterized by heightened detrusor contractility and increased micturition frequency, which serves to compensate for the elevated urine output resulting from osmotic diuresis. In the intermediate phase (weeks 5–8), early decompensatory changes emerge, featuring impaired bladder sensation, a gradual rise in residual urine volume, and declining detrusor contractility. This period is critical for the appearance of typical DNB symptoms. In the late stage (≥9 weeks), the bladder enters a phase of full decompensation, presenting with significant bladder distension, increased compliance, markedly reduced contractility, and severe manifestations such as urinary retention (2).

The 8-week time point corresponds to the transition from compensatory to decompensatory phases. At this stage, characteristic DNB pathological alterations are clearly observable, yet the bladder has not yet progressed into the irreversible severe decompensatory phase, rendering it highly suitable for mechanistic investigations, particularly those assessing the efficacy of therapeutic interventions. Moreover, the average duration for diabetic patients to develop significant bladder dysfunction is approximately 5–10 years, and the pathological changes observed in rodents at 8 weeks closely resemble those in humans at this disease stage, thereby enhancing the translational relevance of the model (40, 41). Additionally, an 8-week modeling period helps avoid increased animal mortality associated with prolonged diabetic conditions.

Further stratified analysis of modeling duration indicated that studies with modeling cycle>12 weeks were significantly more likely to report bladder fibrotic biomarkers (collagen deposition, fibrosis-related mRNA overexpression) as primary detection indicators, whereas studies within 5–8 weeks mostly focused on functional urodynamic and oxidative stress markers. This data-driven distinction further supports 8-week as optimal timepoint for early functional research and>12w for advanced fibrotic pathological observation.

It is worth noting that the transition from detrusor overactivity to underactivity, which mirrors the prevailing theory of human DNB progression, is not uniformly observed across STZ-induced models. While very few studies report a biphasic urodynamic pattern, others document only an underactive or overactive phenotype depending on the duration of diabetes and the specific induction protocol. Future studies should systematically characterize and report the temporal urodynamic phenotype to clarify the proportion of models that recapitulate this clinically relevant transition. Meanwhile, unlike inherited diabetic rodent models that spontaneously develop hyperglycemia and progressive bladder lesions driven by inherent gene mutation, STZ exerts off-target biological effects including direct NLRP3 inflammasome activation within bladder urothelial cells independent of persistent hyperglycemia, which may induce isolated inflammatory bladder damage and interfere with the authenticity of neurogenic pathological changes (36). Such non-specific toxic effect is an inherent limitation of STZ-induced modeling.

### 4.4 Selection and considerations for positive control agents

In scientific research, positive control agents serve as benchmark references to validate experimental systems, enhance the reliability of outcomes, and support decision-making in drug development. Their use is particularly critical in areas such as DND, where the complex pathogenesis—involving multiple molecular pathways—has thus far hindered the development of specifically targeted therapies. Current management strategies primarily focus on glycemic control using oral hypoglycemic agents or insulin, supplemented by neurotrophic support, antioxidative stress interventions, or receptor-targeted drugs that improve detrusor contractility (42).

A key challenge in translating findings across species arises from anatomical, metabolic, and genetic differences, which significantly influence drug pharmacokinetics and pharmacodynamic responses. In the present analysis, the majority of studies employed rodent models, with limited use of other species such as New Zealand rabbits. This narrow taxonomic focus may obscure species-specific drug response patterns, thereby limiting the generalizability of positive control effects. Furthermore, attributing model-specific traits to universal drug mechanisms without cross-species validation can lead to misinterpretation of therapeutic actions. It is therefore essential that future DNB research incorporates a broader range of animal species and conducts detailed mechanistic comparisons to improve the translational relevance of positive control agents and clarify their efficacy across different biological contexts.

### 4.5 Criteria for model validation

The successful establishment of a DNB animal model is characterized by persistent hyperglycemia, rendering blood glucose levels a fundamental criterion for validating model induction. Commonly applied diagnostic thresholds include fasting or random blood glucose concentrations exceeding 11.1 (≈200 mg/dL) or 16.7 mmol/L (≈300 mg/dL). Concurrently, bladder dysfunction—a hallmark of DND—can be objectively quantified through urodynamic assessments, which serve as a core indicator of lower urinary tract pathology.

Urodynamic evaluation represents a critical diagnostic approach for functionally characterizing the lower urinary tract. By measuring parameters such as intravesical and sphincter pressures, urinary flow rates, and bladder sensation, this methodology plays an essential role in identifying and managing neurogenic lower urinary tract dysfunction (43). Rather than a single test, urodynamics comprises a standardized multistep procedure, including free uroflowmetry, postvoid residual urine measurement, filling cystometry (assessing bladder sensation, capacity, compliance, and detrusor activity), pressure–flow studies (evaluating voiding efficiency), and occasionally urethral pressure profilometry or electromyography (43, 44).

However, conventional urodynamic protocols present several limitations, including their invasive nature, nonphysiological testing conditions, lack of operational standardization, and interobserver variability in interpretation (45, 46). In animal studies, invasive catheterization can induce bladder trauma and discomfort, potentially confounding subsequent experimental outcomes. Consequently, not all studies incorporate urodynamics as a primary endpoint. Similarly, histopathological examinations of bladder tissue or measurements of bladder wet weight can only be performed postmortem, restricting their utility for longitudinal assessment.

Given these constraints, there is growing interest in noninvasive alternatives for model validation, such as ultrasonographic imaging and implantable wireless sensors, which may serve as future criteria for evaluating DNB models while minimizing animal distress (47–49).

According to our pooled statistical results, we propose a unified three-in-one minimal validation protocol for future DNB induced animal models: ①metabolic indicator: fasting blood glucose (with matched HbA1c if feasible); ②functional indicator: core urodynamic parameters (bladder capacity + residual urine); ③histological indicator: routine bladder HE or Masson staining for pathological lesion confirmation, which integrates metabolic, functional and structural dimensions to avoid single-index validation bias.

### 4.6 Detection indicators

The evaluation framework employed in DND-related research is structured around a dual-focused approach, addressing both systemic diabetic pathophysiology and localized bladder pathology. Key indicators include measures of blood glucose and insulin activity to elucidate the underlying metabolic disturbances, complemented by bladder-specific assessments—such as histochemical staining and urodynamic profiling—that together provide a multidimensional evaluation of structural and functional integrity.

Methodologically, the detection system integrates conventional morphological techniques with contemporary molecular biology assays. Morphological analyses deliver visual evidence of tissue alterations, while methods including Western blot, immunohistochemistry (IHC), and reverse transcription quantitative polymerase chain reaction (RT-qPCR) establish a robust “transcription–translation” evidence pathway. This approach is further strengthened by biochemical profiling and functional validations, ensuring comprehensive coverage.

In terms of organ involvement, the detection panel systematically captures interorgan interactions involving the bladder, nervous system, kidneys, and pancreatic islets. While the bladder–nerve axis remains the primary focus, the indicators also account for the frequent coexistence of diabetic nephropathy and islet dysfunction.

The core pathogenic mechanisms of DNB are intertwined rather than sequentially linear: oxidative stress serves as an upstream initiator to trigger NLRP3 inflammasome activation and subsequent inflammatory response via elevated IL−1β and other pro-inflammatory cytokines; oxidative stress and inflammatory mediators collectively induce peripheral neuroinjury and parenchymal cell apoptosis. Chronic inflammation further promotes bladder interstitial fibrosis, while fibrosis itself is not a primary inducer of neural damage. Collectively, oxidative stress, inflammation, neurodamage, fibrosis and cell death constitute an interactive pathological network of diabetic bladder injury.

Overall, this detection system adheres to a logical sequence spanning “etiology → core pathology → key mechanisms → clinical translation,” effectively bridging basic and clinical research domains. It combines morphological and functional insights, aligns molecular findings with macroscopic phenotypes, and reflects the multisystem nature of DND, all within a standardized biomedical research framework.

### 4.7 Limitations

This data mining study has several inherent limitations. First, our literature retrieval was restricted to seven mainstream Chinese and English databases, which may lead to potential retrieval bias and incomplete inclusion of relevant studies published in other regional databases or non-English journals. Second, we could only extract summarized aggregate data from the included articles, and raw individual animal experimental data were unavailable. Accordingly, further quantitative meta-analysis to pool effect sizes was not feasible. Third, the retrieved literature was overwhelmingly dominated by chemically induced DNB models. Genetically spontaneous diabetic models accounted for a very low proportion, so we could not conduct quantitative phenotypic comparisons between induced and inherited models. Additionally, STZ exerts direct off-target effects on bladder tissues by activating the NLRP3 inflammasome independent of hyperglycemia, which means STZ-induced models cannot fully recapitulate the natural pathological progression of clinical DND.

Apart from the limitations of the present analysis, current preclinical studies on DNB also exhibit widespread methodological shortcomings. Most studies rely heavily on glycemic indicators to confirm successful model establishment, and many use blood glucose as the sole validation index, which fails to distinguish general diabetic lesions from DND-specific pathological changes. Objective quantitative metrics for evaluating the overall physical status of experimental animals are also rarely reported. Although urodynamics serves as a core functional detection method, its application is limited by invasiveness and inter-operator variability. Moreover, existing studies mainly classify type 1 and type 2 DNB merely according to induction approaches, rather than comprehensive pathological characteristics such as neuropathy and detrusor dysfunction, resulting in a certain degree of disconnection between animal models and clinical manifestations and reducing translational potential. In addition, experimental designs and reporting specifications vary greatly across studies, and incomplete descriptions of randomization, blinding, baseline animal information and housing conditions further increase methodological bias.

To improve the standardization and reproducibility of future studies, we propose a minimum reporting checklist for DNB animal models. Researchers should fully document animal strain, sex, age and body weight, as well as exact drug dosage, administration route, detailed dietary composition and unified modeling duration. We also recommend adopting a multi-dimensional validation system containing at least one metabolic, one functional and one histopathological indicator. Furthermore, complete descriptions of randomization and blinding procedures are required to enhance methodological rigor.

## 5 Conclusion

This study addresses a significant gap in the existing literature by providing a quantitative, evidence-based summary of DNB modeling practices, thereby offering a foundational reference for standardizing future animal experiments. However, the analysis also identified several recurring methodological limitations in the current body of research, including insufficient implementation of blinding procedures and incomplete reporting of baseline animal characteristics. To enhance the quality, reliability, and clinical translatability of future research, efforts should be directed towards refining modeling protocols, incorporating a wider range of animal species in study designs, promoting the use of non-invasive detection technologies such as ultrasonography, and improving the overall methodological rigor in reporting.

## Data Availability

The original contributions presented in the study are included in the article/**Supplementary Material**. Further inquiries can be directed to the corresponding author.
