# Supplementary material for: Induced diabetic neurogenic bladder animal models: application characteristics and standardization proposals via systematic data mining
Source: Front Endocrinol (Lausanne). 2026 Jul 7;17:1804610. doi: 10.3389/fendo.2026.1804610 (PMC13385230; doi:10.3389/fendo.2026.1804610)
Supplement: Supplementary file 1 [file DataSheet1.docx]

**Table S1** DNB modeling methods and their frequencies.

| Category | Specific Classification | Frequency | Proportions (%) |
| --- | --- | --- | --- |
| Chemically induced | STZ | 261 | 74.15 |
|  | Alloxan | 3 | 0.85 |
|  | Total | 264 | 75.00 |
| Combination induced | STZ + high-sugar and high-fat diet | 48 | 13.64 |
|  | STZ + high-fat diet | 32 | 9.09 |
|  | STZ + high-sugar diet | 3 | 0.85 |
|  | STZ + high-salt diet | 1 | 0.28 |
|  | Spontaneous + high-fat diet | 1 | 0.28 |
|  | Total | 85 | 24.15 |
| Not specified |  | 2 | 0.57 |
| Diet-induced | High-sugar and high-fat diet | 1 | 0.28 |

**Table S2** Different injection dose and times of different injection methods.

| Modeling Method | Injection Method | Dose (mg/kg) | Times of Injections (only applicable to multiple injections) | Frequency | Proportions (%) |
| --- | --- | --- | --- | --- | --- |
| STZ | Single injection | 60 mg/kg |  | 87 | 25.07% |
|  |  | 55 mg/kg |  | 42 | 12.10% |
|  |  | 65 mg/kg |  | 39 | 11.24% |
|  |  | 50 mg/kg |  | 24 | 6.92% |
|  |  | 200 mg/kg |  | 16 | 4.61% |
|  |  | 58 mg/kg |  | 11 | 3.17% |
|  |  | Not specified |  | 5 | 1.44% |
|  |  | 150 mg/kg |  | 2 | 0.58% |
|  |  | 62 mg/kg |  | 2 | 0.58% |
|  |  | 100 mg/kg |  | 1 | 0.29% |
|  |  | 35 mg/kg |  | 1 | 0.29% |
|  |  | 40 mg/kg |  | 1 | 0.29% |
|  |  | 45 mg/kg |  | 1 | 0.29% |
|  | Subtotal |  |  | 232 | 66.86% |
|  | Multiple injections | 50 mg/kg | 5 | 24 | 6.92% |
|  |  | 45 mg/kg | 5 | 2 | 0.58% |
|  |  | 55 mg/kg | 5 | 1 | 0.29% |
|  |  | 65 mg/kg | 5 | 1 | 0.29% |
|  | Subtotal |  |  | 28 | 8.07% |
|  | Not specified |  |  | 1 | 0.29% |
|  | Total |  |  | 260 | 74.93% |
| STZ + high-sugar and high-fat diet | Single injection | 50 mg/kg |  | 4 | 1.15% |
|  |  | 30 mg/kg |  | 3 | 0.86% |
|  |  | Not specified |  | 2 | 0.58% |
|  |  | 200 mg/kg |  | 1 | 0.29% |
|  |  | 35 mg/kg |  | 1 | 0.29% |
|  |  | 45 mg/kg |  | 1 | 0.29% |
|  |  | 70 mg/kg |  | 1 | 0.29% |
|  | Subtotal |  |  | 13 | 3.75% |
|  | Multiple injections | 30 mg/kg | 3 | 10 | 2.88% |
|  |  | 40 mg/kg | 5 | 8 | 2.31% |
|  |  | 35 mg/kg | 5 | 4 | 1.15% |
|  |  | 90 mg/kg, 25~30 mg/kg | 2 | 4 | 1.15% |
|  |  | 60 mg/kg, 30 mg/kg | 2 | 3 | 0.86% |
|  |  | 32 mg/kg | 5 | 2 | 0.58% |
|  |  | 33 mg/kg | 5 | 1 | 0.29% |
|  |  | 38 mg/kg | 5 | 1 | 0.29% |
|  | Subtotal |  |  | 33 | 9.51% |
|  | Not specified |  |  | 2 | 0.00% |
|  | Total |  |  | 48 | 0.58% |
| STZ + high-fat diet | Single injection | 200 mg/kg |  | 2 | 13.83% |
|  |  | 120 mg/kg |  | 1 | 0.58% |
|  |  | 30 mg/kg |  | 1 | 0.29% |
|  |  | 35 mg/kg |  | 1 | 0.29% |
|  |  | 50 mg/kg |  | 1 | 0.29% |
|  | Subtotal |  |  | 6 | 0.29% |
|  | Multiple injections | 30 mg/kg | 5 | 14 | 1.73% |
|  |  | 40 mg/kg | 5 | 3 | 4.03% |
|  |  | 32 mg/kg | 5 | 2 | 0.86% |
|  |  | 35 mg/kg | 5 | 2 | 0.58% |
|  |  | 50 mg/kg | 5 | 2 | 0.58% |
|  |  | 25 mg/kg | 5 | 1 | 0.58% |
|  |  | 45 mg/kg | 5 | 1 | 0.29% |
|  |  | Not specified | 2 | 1 | 0.29% |
|  | Subtotal |  |  | 26 | 0.29% |
|  | Total |  |  | 32 | 7.49% |
| STZ + high-sugar diet | Not specified |  |  | 2 | 9.22% |
|  | Single injection | 200 mg/kg |  | 1 | 0.58% |
|  | Total |  |  | 3 | 0.29% |
| Alloxan | Single injection | 250 mg/kg |  | 3 | 0.86% |
| STZ + high-salt diet | Single injection | 50 mg/kg |  | 1 | 0.86% |

**Table S3** Methods of dietary induction.

| Diet-induced | Specific Time and Method | Frequency | Proportions (%) |
| --- | --- | --- | --- |
| High-sugar and high-fat diet | Ad libitum feeding | 32 | 37.21 |
|  | After weaning | 6 | 6.98 |
|  | STZ injection after 4 weeks of feeding | 3 | 3.49 |
|  | From 4 weeks of age | 3 | 3.49 |
|  | 2 weeks after the last STZ injection | 2 | 2.33 |
|  | From 5 weeks of age | 1 | 1.16 |
|  | Start feeding after weaning | 1 | 1.16 |
|  | Feed for 3 weeks before modeling | 1 | 1.16 |
|  | Total | 49 | 56.98 |
| High-fat diet | Ad libitum feeding | 32 | 37.21 |
|  | From 5 weeks of age | 1 | 1.16 |
|  | Total | 33 | 38.37 |
| High-sugar diet | Ad libitum feeding | 2 | 2.33 |
|  | STZ injection after 1 month of feeding | 1 | 1.16 |
|  | Total | 3 | 3.49 |
| High-salt diet | Ad libitum feeding | 1 | 1.16 |

**Table S4** Modeling criteria and its frequency.

| No. | Modeling Criteria | Frequency | Proportions (%) |
| --- | --- | --- | --- |
| 1 | Blood glucose (fasting or random blood glucose ≥ 11.1 mmol/L, fasting or random blood glucose ≥ 16.7 mmol/L, etc.) | 345 | 52.11 |
| 2 | Urodynamics (measurement of maximum bladder pressure, maximum bladder capacity, leak point pressure, resting intravesical pressure, bladder compliance, residual urine volume, etc.) | 81 | 12.24 |
| 3 | Glycated hemoglobin (HbA1c) (HbA1c ≥ 6.5%, HbA1c > 6.8%, HbA1c ≥ 7.0%, etc.) | 38 | 5.74 |
| 4 | Bladder tissue pathology (HE staining, VG staining, Masson staining, etc., showing disordered arrangement of detrusor muscle fibers, hypertrophy of detrusor cells, significant increase in collagen fibers, enlarged intermuscular spaces, etc.) | 21 | 3.17 |
| 5 | Insulin sensitivity (insulin resistance index, insulin sensitivity, etc.) | 19 | 2.87 |
| 6 | Bladder wet weight (higher than that of the normal group) | 13 | 1.96 |
| 7 | Glucose tolerance (2-hour postprandial blood glucose > 11.9 mmol/L, postprandial blood glucose ≥ 7.8 mmol/L, increased area under the glucose tolerance test curve, etc.) | 13 | 1.96 |
| 8 | General conditions (loss of body mass, lack of luster of the animal’s coat, increased water intake, dry and loose stools, etc.) | 12 | 1.81 |
| 9 | Urine glucose (urine glucose +++, ++++) | 11 | 1.66 |
| 10 | Urine volume (24-hour urine volume, increased urine volume) | 9 | 1.36 |
| 11 | Urinary microalbumin (increased urinary microalbumin, ≥ 2-fold increase) | 9 | 1.36 |
| 12 | Insulin (decreased pancreatic insulin content > 45%, decreased insulin secretion > 40%, etc.) | 8 | 1.21 |
| 13 | Creatinine (Urinary Albumin-to-Creatinine Ratio (UACR) > 30 mg/g, > 40 mg/g, 50 mg/g, etc.) | 7 | 1.06 |
| 14 | Voiding frequency (increased voiding frequency > 30%, > 1.5 times per hour in the awake state, etc.) | 7 | 1.06 |
| 15 | Body weight (significant weight loss or lower than that of the normal group) | 7 | 1.06 |
| 16 | Pancreatic β-cells (decreased number of pancreatic β-cells > 40%, increased apoptosis rate > 45%, etc.) | 6 | 0.91 |
| 17 | Isolated detrusor muscle experiment (contractile stimulation test of isolated detrusor muscle rings, mechanical stretch test of isolated detrusor muscle strips, electrical stimulation test of isolated detrusor muscle strips, etc.) | 5 | 0.76 |
| 18 | Renal tissue pathology (renal pathological changes, renal inflammatory response, etc.) | 5 | 0.76 |
| 19 | Lower urinary tract symptom (LUTS) score (increased LUTS score > 50% or > 60%, increased lower urinary tract obstruction (LUTO) score > 50%, etc.) | 4 | 0.60 |
| 20 | Insulin tolerance test (ITT) (decreased blood glucose decline rate in ITT, decreased blood glucose clearance rate > 25%) | 4 | 0.60 |
| 21 | Neuroelectrophysiological examination (abnormal neurotransmitters in bladder tissue, decreased contractile amplitude under electric field stimulation > 30%, nerve conduction velocity, etc.) | 3 | 0.45 |
| 22 | Others (urine osmolality, delayed micturition reflex, etc.) | 35 | 5.29 |

**Table S5** Results of association rule analysis of DNB animal model formation criteria.

| Consequent | Antecedent | Support Percentage | Confidence Percentage | Instances | Gain |
| --- | --- | --- | --- | --- | --- |
| A | B | 23.01 | 95.06 | 81 | 0.97 |
| A | C | 10.80 | 100.00 | 38 | 1.02 |
| A | D | 5.97 | 95.24 | 21 | 0.97 |
| B | D | 5.97 | 52.38 | 21 | 2.28 |
| B | D and A | 5.68 | 50.00 | 20 | 2.17 |
| A | E | 5.40 | 100.00 | 19 | 1.02 |
| A | F | 3.69 | 100.00 | 13 | 1.02 |
| A | G | 3.69 | 84.62 | 13 | 0.86 |
| B | F | 3.69 | 61.54 | 13 | 2.67 |
| B | F and A | 3.69 | 61.54 | 13 | 2.67 |
| A | H | 3.41 | 91.67 | 12 | 0.94 |
| J | H | 3.41 | 50.00 | 12 | 19.56 |
| A | I | 3.13 | 100.00 | 11 | 1.02 |
| A | D and B | 3.13 | 90.91 | 11 | 0.93 |
| J | H and A | 3.13 | 54.55 | 11 | 21.33 |

**Note:** Visualization network diagram of molding criteria correlation analysis. A: Blood glucose; B: Urodynamics; C: Glycated hemoglobin; D: Bladder tissue pathology; E: Insulin sensitivity; F: Bladder wet weight; G: Glucose tolerance; H: General conditions; I: Urine glucose; J: Urine volume.

**Table S6** Frequency distribution of detection indicators.

| Detection indicators | Specific indicators | Frequency | Proportions (%) |
| --- | --- | --- | --- |
| Blood Glucose and Insulin | Blood Glucose, Fasting Blood Glucose, 2-Hour Postprandial Blood Glucose, Random Blood Glucose, Insulin Release Test, Insulin Tolerance Test, Homeostasis Model Assessment of Insulin Resistance, etc | 305 | 18.37% |
| Histochemical Staining | Bladder tissue morphology, Arrangement of detrusor muscle fibers in the bladder, Morphological structure of detrusor cells, Thickness of the muscle layer in the bladder wall, Smooth muscle cell density, Integrity of the epithelial tissue in the bladder, Degree of fibrosis in the smooth muscle layer, Number of inflammatory cell infiltrates, etc | 264 | 15.90% |
| General Condition | Body Weight, Feed Intake, Water Intake, Urine Output, Mental State, Hair Coat Luster, Survival Status, Body Length, etc | 195 | 11.75% |
| Western blot | α-SMA、TGF-β1、NF-κB p65、E-cadherin、p-Smad2/Smad2、Col-IV、p-Smad3/Smad3、α1-AR、α1A-AR、α1A-AR mRNA、α1D-AR、ZO-1、Occludin、VEGF、VEGFR2、HIF-1α、UCP1、TRPV4、TNF-α、IL-1β、TrkA、p75NTR、TLR4、p-NF-κB p65、IL-6、MyD88、NLRP3、caspase-1、IL-18、ASC、caspase-1 p20、Smad3、MMP-13、TIMP-1、Col-I、Col-III、GAPDH、CTGF、Synapsin I、PSD-95、SIRT3、Ac-SOD2、Drp1、Mfn2、p-Smad3、SIRT1、p53、Ac-p53、p-PI3K、PI3K、p-Akt、Akt、PPAR-γ、AMPKα、AdipoQ、podocin、nephrin、WT1、p-mTOR、mTOR、p-4EBP1、4EBP1、LC3、p62、Beclin-1、p-MLCK、p-MLC、PKM、Snail1、Twist1、Nrf2、HO-1、NQO1、Keap1、iNOS、nNOS、ChAT、NKCC2、ROMK1、BDNF、MBP、IκBα、NF200、Syn、NGF、mAChR M3、P2X7、mAChR M2、Ki-67、Cyclin D1、IRS-1、CCL2、CXCL1、p-IκBα、HSP27、HSP27 (Ser15/Ser78/Ser82)、CaD、TM、GSH-Px、GR、T-AOC、8-OHdG、GSH、GPx、GSDMD-N、caspase-4、caspase-11、GRP78、CHOP、caspase-12、GR、4-HNE、SOD1、GPX4、ACSL4、Ferroportin、ERS、eNOS、Bcl-2、Bax、DRG、P2X3、TRPV1、P2X2、NPY、Nav1.8、Nav1.9、c-Kit、TH、AChE、cAMP、cTnT、MHC、Cyt C、FN、AMPK、p-AMPK、p-Akt/Akt、LC3-II/LC3-I | 176 | 10.60% |
| Immunohistochemistry | α-SMA、BDNF、NGF、FN、MBP、Islet β-cell、PGP9.5、NF-200、NOS、ChAT、8-OHdG、ACSL4、Akt、AQP5、ASC、Bax、Bcl-2、Beclin-1、Ca²⁺-ATPase、CaD、caspase-1、Caspase-3、caspase-4、CD31、CD68、CGRP、CHOP、c-kit、claudin-1、ColI、ColIII、ColIV、COXIV、CTGF、cTnT、Cx43、CyclinD1、DRG、E-cadherin、EGFP、eNOS、GFAP、GLUT2、GLUT4、GPX4、GR、GRP78、GSDMD-N、GSH-Px、HIF-1α、HO-1、HSP27、Iba1、IGF-1、IL-18、IL-1β、IL-6、iNOS、Ki-67、Kim-1、LC3、Ly6G、M3mAChR、M3 receptor、MHC、MMP-2、mPGES-1、Na⁺/K⁺-ATPase α1 subunit、Co-localization of Nav1.8/Nav1.9 and NF200、NF、NF-κB、NF-κBp65、NHE3、NLRP3、nNOS、NPY、NQO1、O-1、Occludin、P2X2、P2X3、P2X7、p-4EBP1、p62、p75、p75NTR、p-Akt、PI3K、p-mTOR、p-PI3K、S100、SOD1、SOD2、Syn、TGF-β、TGF-β1、TH、TM、TNF-α、TOPRO3、TrkA、TRPV1、TRPV4、VAT、VCAM-1、VEGF、VIP、vWF、ZO-1、α₁A、α1A-AR、α1-AR、α₁D、Bladder wall nerve fiber density、Testosterone、Keratin 18、Nitrosative stress marker、Nitrotyrosine、Astrocyte activation、Positive cell count、Number of cholinergic neurons in the anterior horn of lumbosacral spinal cord、Insulin、Insulin antibody、Acetylcholinesterase、Podocyte | 161 | 9.70% |
| RT-qPCR | M3R mRNA、nNOS mRNA、ChAT mRNA、TNF-α mRNA、IL-6 mRNA、VEGF、HIF-1α mRNA、AMPK、mTOR、Beclin-1、LC3、SIRT1、PGC-1α、TGF-β1 mRNA、Smad2、ACC、SREBP-1c、Bax、Bcl-2、NF-κB、p-AKT、α-SMA、FN、caspase-3、BDNF mRNA、cAMP、BNP mRNA、TH、c-kit、c-kit mRNA、Col1a1、Col3a1、cTnT、MHC、Cyclin D1、DRG、eNOS mRNA、Fura-2/AM、GRP78、CHOP、Caspase-12 mRNA、HO-1、NQO1、HSP27 mRNA、CaD mRNA、TM、IL-10、CCL2、CXCL1、iNOS、LH-R、FSH-R、LPS、M2R、M2R mRNA、M3R、P2X7、MCP-1、MDA、mtDNA、Nav1.8、Nav1.9、NGF mRNA、FoxO1、MBP、p75NTR、NHE3、Na⁺/K⁺-ATPase α1 subunit、NKCC2、ROMK1、NLRP3、ASC、IL-18、NF-κB p65、Keap1、P2X3、TRPV1 mRNA、PI3K mRNA、AKT mRNA、Snail1、Twist1、E-cadherin、Piezo1 mRNA、Piezo2 mRNA、PKM、podocin、nephrin、WT1、PPAR-α、CPT-1α、FAS mRNA、PPAR-γ mRNA、C/EBPα mRNA、ROCK1、ROCK2、SCF mRNA、SOD2、Drp1、Mfn2、CTGF、Col-IV、TLR4、MyD88、IL-1β mRNA、α1A-AR mRNA、α1B-AR mRNA、α1D-AR mRNA、α1-AR、SMMHC mRNA、β1-AR mRNA、β3-AR、ICCs (c-kit⁺) density in detrusor muscle、Gja1 mRNA（Cx43 mRNA）、SCL mRNA、IL-6/TNF-α mRNA（liver）、AVP V2 receptor mRNA、TNF-α/IL-6 mRNA（kidney）、Insulin gene、TNF-α/IL-6 mRNA（pancreas） | 142 | 8.55% |
| Urodynamic Indicators | Maximum Voiding Pressure, Maximum Urinary Flow Rate, Residual Urine Volume, Bladder Compliance, Bladder Contraction Duration, etc. | 124 | 7.47% |
| Elisa | IL-1β、IL-6、IL-18、TNF-α、AGE、MDA、ROS、GSH、AVP、cAMP、caspase-3、CRP、IgG、iNOS、LH、LPS、NGF、PACAP38、PGE₂、ROCK1、ROCK2、p-PI3K、p-Akt、TrkB、ZO-1、Occludin、SP、TG、TC、T、Ins、Pancreatic insulin secretion、Colonic tissue inflammation score、Rat liver mitochondria | 89 | 5.36% |
| Transmission Electron Microscopy | Ultrastructure of Bladder Tissue (Mucosal Layer, Submucosal Layer, Nervous Tissue, Smooth Muscle Cells, etc.), Ultrastructure of Interstitial Cells of Cajal (ICC), Ultrastructure of Pancreatic β-Cells, Ultrastructure of Spinal Dorsal Root Ganglia, etc | 80 | 4.82% |
| Bladder-Related test | Bladder Wet Weight, Bladder Wall Thickness, Bladder Wet Weight/Body Weight Ratio, Isolated Bladder Perfusion Experiment, Relaxation Response of Bladder Tissue to β-Adrenergic Agonists, Incidence of Detrusor Instability, etc | 61 | 3.67% |
| TUNEL Assay | Bladder Smooth Muscle Cell Apoptosis Rate, Bladder Epithelial Cell Apoptosis Rate, Bladder Tissue Cell Apoptosis Rate, Pancreatic β-Cell Apoptosis Rate, Pancreatic β-Cell Pyroptosis Rate, Bladder Endothelial Cell Apoptosis Rate, Bladder Nerve Cell Apoptosis Rate, Renal Tubular Epithelial Cell Apoptosis Rate, etc | 35 | 2.11% |
| Others | Colorimetric Assay (for Iron Ion Concentration, Superoxide Dismutase, etc.), Thiobarbituric Acid Assay (for Malondialdehyde, MDA), Northern Blotting (for Content of Neuronal Nissl Bodies), Detrusor Strip Contraction-Relaxation Test, etc. | 20 | 1.20% |
| Renal Function | Urine Microalbumin/Creatinine Ratio, Blood Urea Nitrogen, Serum Creatinine, Glomerular Filtration Rate, Renal Plasma Flow, etc | 8 | 0.48% |


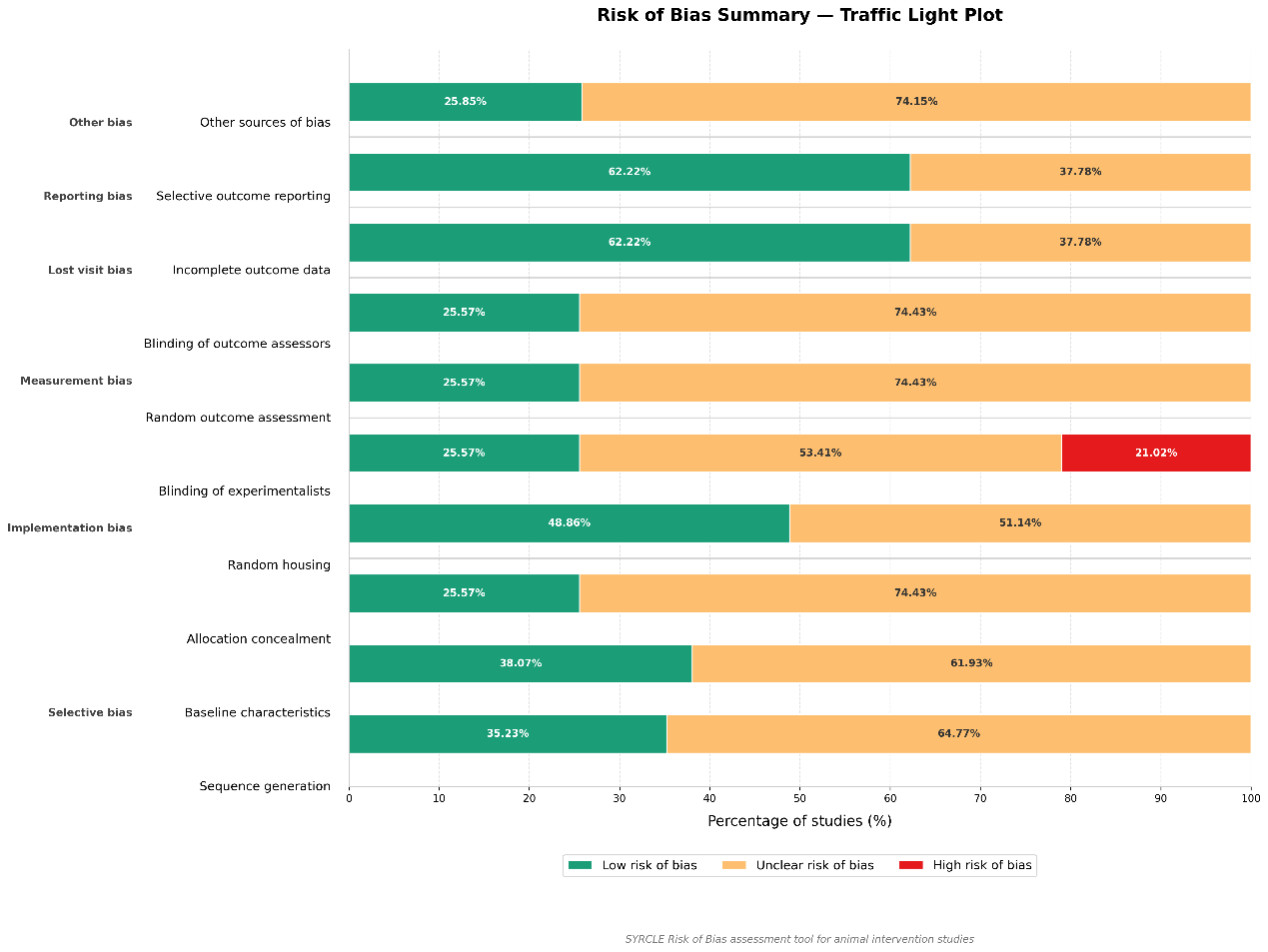


**Figure S1** SYRCLE risk-of-bias assessment of included studies across six evaluation domains (green: low risk; yellow: unclear risk; red: high risk)
